# Therapeutic device as a facilitating resource for esophageal voice acquisition

**DOI:** 10.1016/j.bjorl.2026.101893

**Published:** 2026-09-10

**Authors:** Vaneli Colombo Rossi, Ana Carolina Constantini, Carlos Takahiro Chone

**Affiliations:** a Universidade Estadual de Campinas (UNICAMP), Department of Otolaryngology Head and Neck, Campinas, SP, Brazil; b Universidade Estadual de Campinas (UNICAMP), Department of Human Development and Rehabilitation, Campinas, São Paulo, Brazil

**Keywords:** Laryngeal cancer, Vocal rehabilitation, Esophageal speech, Speech-language pathology, Voice

## Abstract

**Objective:**

To describe the findings on the use of a therapeutic device for direct esophageal stimulation, which facilitates the production of the esophageal voice.

**Method:**

Fifteen total laryngectomized subjects, with no voice production and no success in traditional speech-language pathology therapies for esophageal voice acquisition, participated. Subjects were evaluated using a specific vocal quality protocol for total laryngectomy, videofluoroscopy, and a vocal self-assessment questionnaire before and after 12 sessions of esophageal stimulation with the device. To stimulate esophageal voice acquisition, direct esophageal stimulation was performed using a catheter with a total length of 50 cm, which was introduced from the nostril until reaching the pharyngoesophageal segment; the other end of the catheter was fixed to the tracheostoma. The intervention occurred over 12 consecutive weeks, without interruption. Descriptive and inferential statistical analysis was performed at a 5% significance level.

**Results:**

Nine (60%) subjects developed effective esophageal voice for communication, while 6 (40%) managed to develop esophageal sound. Auditory-perceptual evaluation did not show statistically significant differences between pre- and post-intervention moments; however, participants’ self-assessment indicated perceived improvement in vocal quality.

**Conclusion:**

Esophageal stimulation with the proposed device was associated with improvements in patients’ self-assessment and enabled a proportion of subjects to achieve functional esophageal voice. The device may represent a feasible therapeutic resource.

**Level of Evidence:**

2B – Prospective interventional study without control group.

## Highlights

• Direct esophageal stimulation aided esophageal voice after laryngectomy.
• Sixty percent achieved fluent, intelligible esophageal voice after training.
• The Blom-Singer® tube was effective as a non-surgical therapeutic resource.
• The method offers a low-cost, accessible option for voice rehabilitation.
• Auditory feedback improved patients’ perception and motivation.

## Introduction

Total laryngectomy remains a treatment option for patients diagnosed with advanced laryngeal cancer. Voice loss directly affects individuals undergoing this surgical procedure. This surgical procedure is highly debilitating, leading to a significant decrease in quality of life and psychosocial impacts.^1^

After the surgical procedure, there are three possibilities for vocal rehabilitation: Tracheoesophageal Voice (TEV) via a voice prosthesis, Electrolarynx (EL), and Esophageal Voice (EV). The latter poses a significant challenge for acquisition, both for speech-language pathologists and for laryngectomized individuals.^1^ EV is the least successful rehabilitation method and requires a strong motivational factor, but it is the least expensive,^2^ which makes it important for the Brazilian context. In Brazil, access to TEV and EL through the Unified Health System (Sistema Único de Saúde ‒ SUS) is quite restricted; only a few major rehabilitation centers offer them, and acquiring them through private services is costly and less accessible to individuals.^3^

Laryngectomized individuals, in the long term, generally prefer to use the EV, as it does not require the use of an external device like the EL or the TEV, the latter of which requires surgery for voice prosthesis placement and constant clinical management.^3^ TEV has a higher success rate, but its high cost can sometimes make its use unfeasible, even in the country's reference centers.^3^ The preference for EV is also related to the absence of the need to use hands to speak, nor hospital management, constant replacement, and other complications associated with TEV.^4^ Thus, in Brazil, EV continues to be the main rehabilitation technique to facilitate communication after total laryngectomy, due to social and economic restrictions. Several variables, such as age and socio-emotional status, as well as anatomical and functional factors, have been associated with success in EV acquisition. The performance of a speech-language professional and adherence to speech therapy are essential, as it is a difficult method to acquire.

Previous studies have described the acquisition of EV through conventional speech-language therapy as a time-consuming and variable process. The first production of understandable esophageal speech is typically achieved within a median of 11-weeks of therapy,^5^ while functional or optimal speech is generally reached within 2- to 6-months, depending on therapy intensity and individual characteristics.^6^, ^7^, ^8^ Faster acquisition has been reported in high-intensity protocols, but these conditions may not reflect routine clinical practice.^8^

The time required for EV acquisition is influenced by factors such as therapy frequency and intensity, as well as patient-related variables including motivation, adherence, and clinical condition.^5^^,^^8^ Despite these findings, there is no universally accepted definition of successful EV acquisition, with studies varying from initial phonatory production to functional communication outcomes.^8^

The literature on speech-language rehabilitation after total laryngectomy is limited and somewhat dated, and although vocal rehabilitation options are well-established in scientific literature and professional practice, specifically the acquisition and use of EV has decreased significantly over the last 40-years, due to the difficulty in acquiring the proposed techniques and the need for a long period of speech therapy. The techniques described in the literature for EV acquisition are subjective, difficult to understand and execute, and consist of guidance on the execution of swallowing, aspiration, and/or injection movements.^4^

Due to the subjectivity and difficulty in executing the existing techniques for EV acquisition, we proposed direct esophageal stimulation through a catheter for the exclusive use of total laryngectomized individuals, which has not previously been used for this purpose. It is a potentially facilitating device for EV acquisition with results measured by objective protocols.

The device used was the Blom-Singer® insufflation tube, developed to assess the resistance of the pharyngoesophageal segment for predicting the development of TEV.^9^ The tube conducts pulmonary air to the pharyngoesophageal segment, exactly like the voice prosthesis, but unlike the prosthesis, which requires a surgical incision, the tube does it via a non-surgical route. The first investigations using the Blom-Singer® insufflation tube were described in 1987, to assess the pharyngoesophageal segment before the use of the tracheoesophageal prosthesis, and the author himself mentions that the tube can be an instrument to assess the esophagus before its insertion and also an indicator of a good prognosis for EV production.^9^ Since then, this tube has been used in other countries only for the purpose of assessing the ability of the pharyngoesophageal segment to simulate mucosal vibration before tracheoesophageal prosthesis placement.^10^, ^11^, ^12^ No studies were found describing its use as a therapeutic resource to stimulate the acquisition of EV. Our proposal is to attribute a new utility to the Blom-Singer® insufflation tube, with direct esophageal stimulation as a facilitating resource for EV acquisition. Studies suggest that tracheoesophageal phonation significantly accelerates the acquisition of EV, from 186-days to 59-days, due to the stimulation of pharyngoesophageal segment vibration and improvement in vocal self-perception.^13^ The hypothesis is that the tube could directly stimulate the pharyngoesophageal segment, just like the voice prosthesis, and also be a facilitating resource in the acquisition of EV.

The tube used during the study allows instantaneous production of esophageal sound, which provides auditory and sensitive feedback to the individual and aids in the understanding and knowledge of EV production. Thus, by hearing the production of esophageal sound in the first moment of stimulation, the understanding of what the expected vocal production is in the rehabilitation process is favored. The airflow injected into the pharyngoesophageal segment, through the tube coupled to the tracheostoma, distends this anatomical region, and the learning process of voice modulation and vibration is facilitated, with an increase in the laryngectomized individual's proprioception regarding the control of the amount of air for vocal production.^14^

Therefore, the aim of this study was to investigate the use of the Blom-Singer® insufflation tube as a therapeutic resource to facilitate the acquisition of esophageal voice in total laryngectomized individuals, and to evaluate its effects using auditory-perceptual assessment and patients’ self-assessment measures.

## Methods

### Study location

The study was conducted at the Radiology Department of the UNICAMP, a comprehensive oncological center that includes both patient care and research.

### Study design

This is a prospective, quantitative intervention study, submitted to and approved by the Research Ethics Committee of the UNICAMP, under opinion number 1.813.841. Individuals were recruited through a national database of oncology patients treated in Brazil. After signing the informed consent form, all subjects underwent a clinical evaluation consisting of: (a) Videofluoroscopy of Swallowing (VFS) to evaluate the pharyngoesophageal segment; (b) Application of the Hilgers vocal quality protocol,^15^ with a free translation into Brazilian Portuguese; (c) Evaluation of the subjects' voice production by three speech-language pathologists; and (d) Vocal self-assessment.

All participants underwent an intervention protocol with esophageal stimulation, using the Blom-Singer® insufflation tube,^9^ once a week for 12-sessions, with each session lasting 30-minutes, comprising 10-minutes of direct stimulation in the esophagus with the tube and the remaining 20-minutes of EV training using traditional methods. At the end of the 12 stimulation sessions, all evaluation procedures were repeated for comparison of results.

### Data analysis

The results underwent descriptive and inferential statistical analysis using the Jamovi software (version 2.3.28). The Shapiro-Wilk test showed that the samples did not follow a normal distribution, and thus, the comparison between the groups was performed using non-parametric statistical tests. Differences in the speech-language evaluation and the self-assessment between the pre- and post-intervention moments were analyzed using the Wilcoxon test. To compare the proportions of participants who showed improvement between the pre- and post-stimulation moments, the participants' evaluations and self-assessment were recoded into two categories: “Improved” (“'Good” or “Moderate” evaluations) and “Did not improve” (“Poor” evaluation), considering the post-intervention moment. For this analysis, given the paired design and the presence of null frequencies at the pre-intervention moment, the two-sided binomial test was chosen. A significance level of 5% was adopted.

### Inclusion criteria

Individuals with tumor stage T4; treated for laryngeal cancer with upfront TL (Total Laryngectomy), without extension to the hypopharynx, without associated neurological deficits, disease-free for at least four years before participating in the study, without anatomical or physiological stenosis or spasm in the pharyngoesophageal segment upon VFS examination, with no EV production and no vocal production with previous speech-language pathology therapy elsewhere, characterizing failure in EV acquisition.

### Exclusion criteria

Individuals with distant metastases, local or regional recurrence; those who underwent radiotherapy or chemoradiotherapy before TL, patients who underwent pharyngolaryngectomy, those who received regional flaps associated with TL, presence of residual disease, feeding via a nasoenteral tube or gastrostomy, individuals with esophageal or TEV.

### Participants selection

A survey of all individuals treated for laryngeal cancer with TL between January 2000 and 2012 in the institution's database was conducted, and 584 individuals were identified. Of these, 248 (42%) were classified as T4 (invasive tumor requiring TL). Of the total T4 sample, 104 (42%) were alive at the time of the study. Among these, 58 (56%) had not undergone vocal rehabilitation, and 14 (24%) of them presented with distant metastases and were excluded.

Forty-four subjects were invited by letter in November 2016 to attend the outpatient clinic to participate in the study. Thirty individuals attended the outpatient clinic, and 15 met all inclusion criteria.

**Fig. 1:**
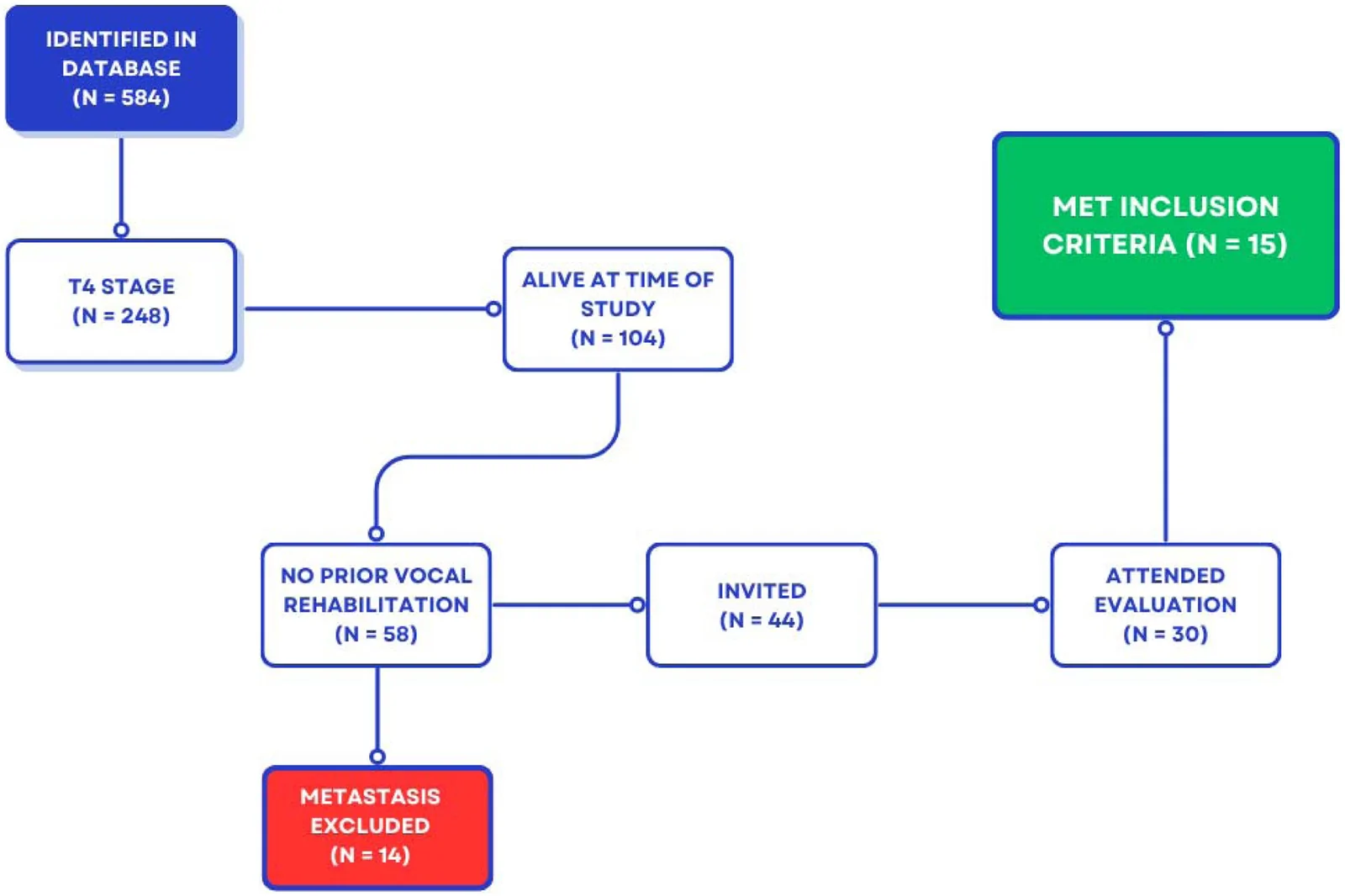
Flowchart of participants selection.

The participant selection process is illustrated in Fig. 1. The flowchart presents the initial number of individuals identified in the institutional database, followed by the application of inclusion and exclusion criteria, and the final sample included in the study.

### Videofluoroscopy

VFS is a moving radiological method that allows for the diagnosis, evaluation, and monitoring of the pharyngoesophageal segment.^16^ It is a dynamic assessment of the anatomy and physiology of swallowing with different consistencies of the oral swallowing test contrasted with barium, mimicking different food consistencies. For the development of the EV, the pharyngoesophageal segment ‒ a structure that plays a fundamental role in swallowing ‒ is used. Therefore, alteration presented during swallowing could prevent the subject from developing the EV. The videofluoroscopic evaluation was performed during swallowing and voice production, in the lateral and anteroposterior planes one week before the start of esophageal stimulation and one week after the end of the 12-intervention sessions. The swallowing analysis was performed using three distinct consistencies: liquid (normal/thin), pasty (nectar/honey), and solid. For the analyses of simulated voice production, subjects were instructed to produce the sustained vowel [a] or the syllable [pa] 10-times, if prolonged phonation was not possible. Additionally, subjects were instructed to count numbers from 1 to 10.

### Hilgers vocal quality protocol

This Global Voice Assessment protocol was developed in 1995 (Annex 1).^15^ It is exclusively designed for the objective assessment of the total laryngectomized patient. The instrument was freely translated into Brazilian Portuguese by the research team and consists of 13-items. Each item is assigned a concept that can vary between good, moderate, or poor. Those who received up to three moderate concepts are judged to have good vocal quality. Those who received one poor concept have moderate vocal quality, and those with two or more poor concepts will be considered to have poor or inadequate vocal quality. This protocol was completed by three speech-language pathologists with experience in the rehabilitation of total laryngectomized individuals before the first stimulation session and repeated after the last stimulation session, based on the participants' voice recordings (procedure described in item 2.6). The three speech-language pathologists had no knowledge about which sample was from the pre- or post-intervention moment. Repeated samples were not considered to test the reliability of the judges (intra- and inter-observer agreement).

### Vocal self-assessment evaluation

To perform the vocal self-assessment evaluation, the participants' voices were recorded in an acoustically treated room at the pre-intervention and post-intervention moments. The principal researcher requested the emission of the following speech tasks at both times: name, age, days of the week, and asked each subject to classify their own voice as good, moderate, or poor. This emission was performed without stimulation of the pharyngoesophageal segment. The recording was done with PRAAT software using the Andrea PureAudio USB Adapter + Karsect HT-9 Microphone interface.

### Blom-Singer® insufflation tube in esophageal stimulation sessions

**Fig. 2:**
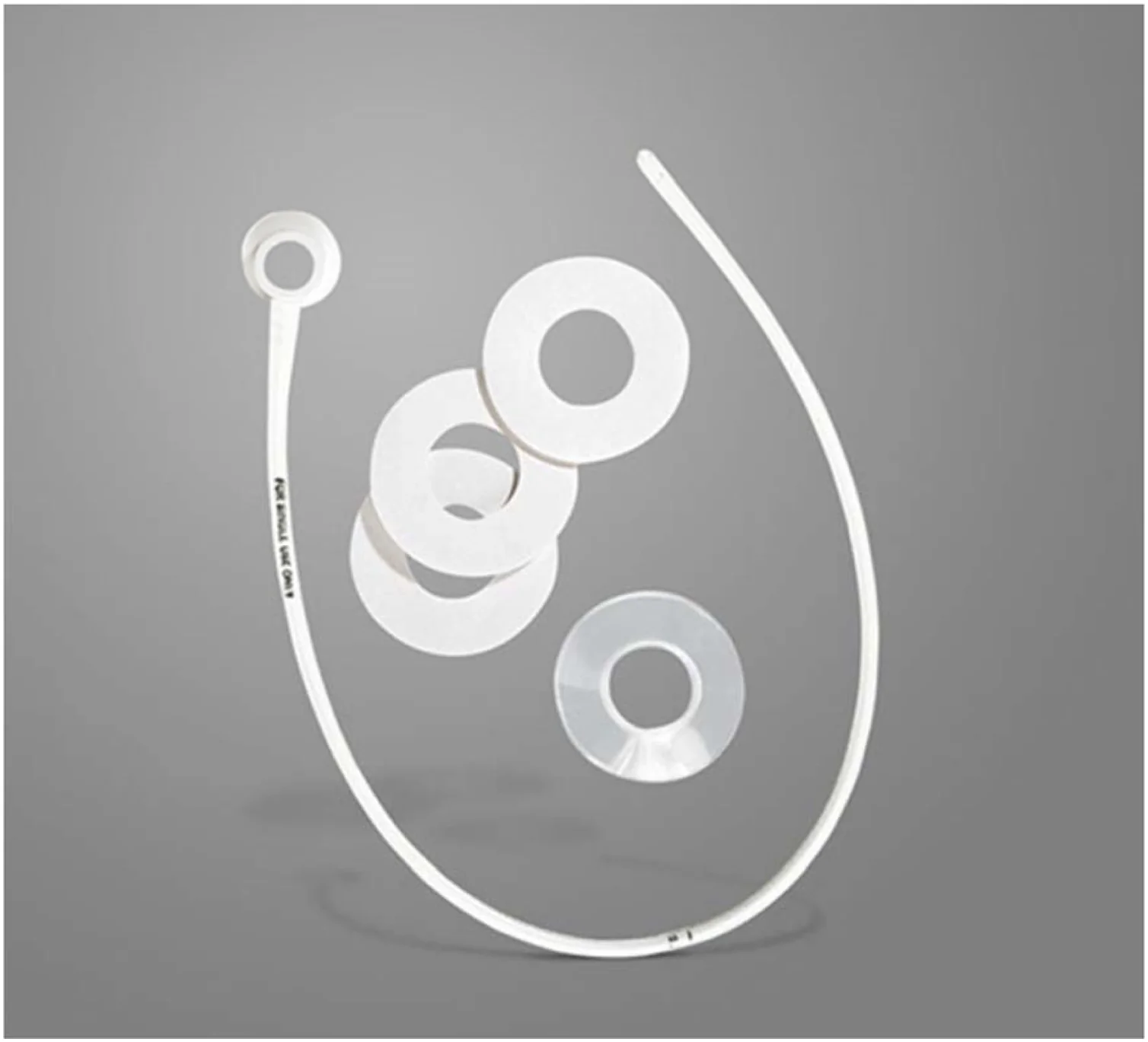
Components of the Blom Singer Insufflation test device.

**Fig. 3:**
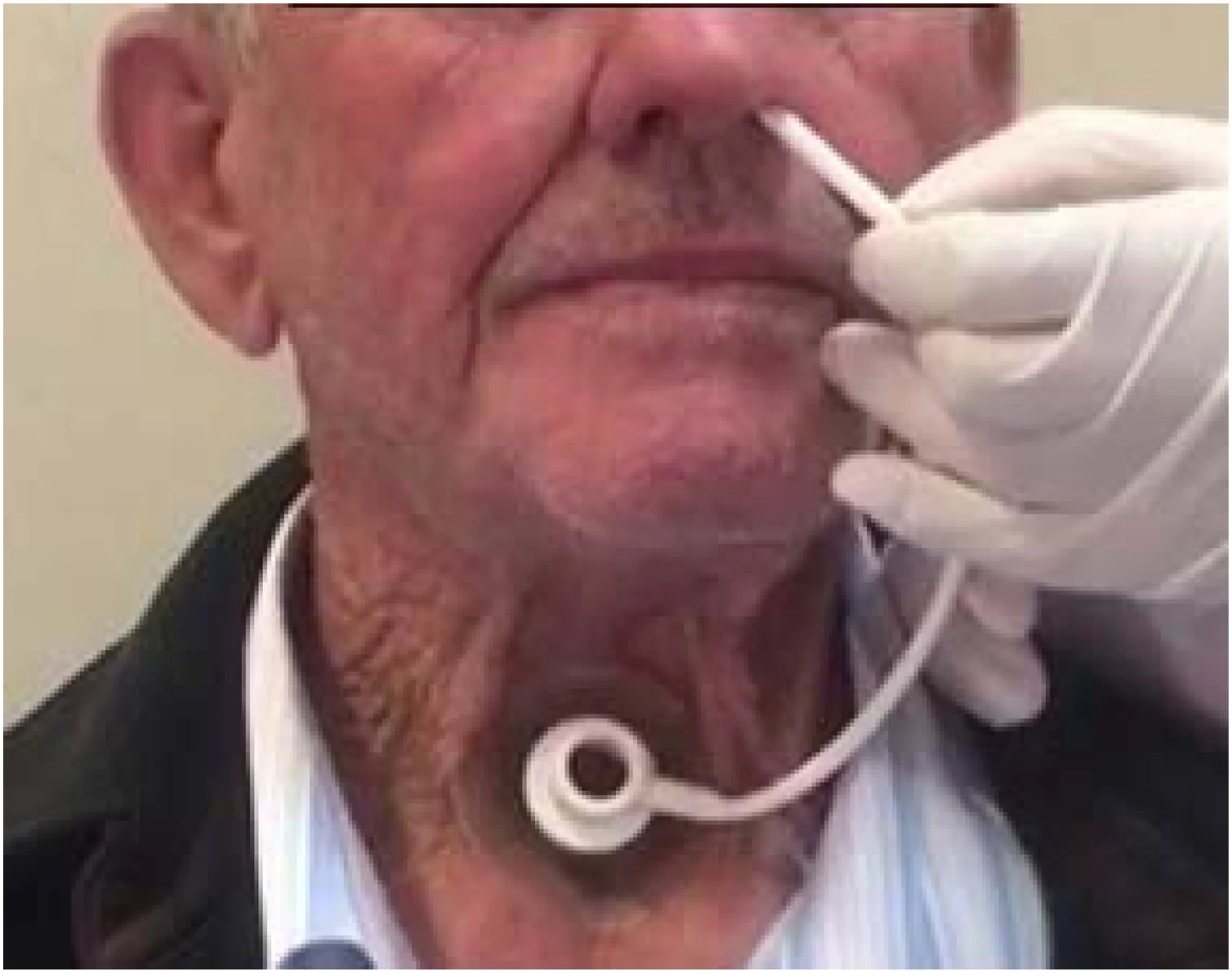
Catheter adapted and fixed.

Esophageal stimulation was performed using the Blom-Singer® insufflation tube,^9^ which consists of a 50-centimeter (cm) long catheter with a tracheostoma adapter at one end and two orifices at the other (Fig. 2). The tracheostoma adapter is fixed to an adhesive indicated for a total laryngectomized stoma, and the other end is introduced 23–25 cm through the nostril.^9^ The end with the 2 parallel orifices must be introduced through the individual's nostril, by a physician or nurse, until it reaches the upper portion of the pharyngoesophageal segment (Fig. 3).

To ensure the tube is in the correct location, after proper placement and fixation, the subject is asked to inhale deeply and produce a sustained vowel [a] upon exhalation. To facilitate the emission, the tracheostoma is occluded, which allows the airflow to be directed into the esophagus through the tube, causing it to vibrate and produce the requested sound. If no sound is emitted, the researcher can slowly withdraw the tube until sound emission occurs. With the production of sound, it is confirmed that the tube is correctly positioned. It can then be secured to the nose with micropore tape to prevent displacement, and the esophageal stimulation training can begin.

For the training of esophageal stimulation production, participants were asked to perform automatic speech tasks such as counting numbers, days of the week, and months of the year, in addition to singing the song “Happy Birthday to you”. This sequence was repeated five times with a ten-second interval between each repetition. After the sequence was completed, a sequence of automatic speech was initiated, such as: asking for full name, place of residence, family composition, work activity, daily life habits, and reading a text. It is important at this moment that the stimulation of the pharyngoesophageal segment with the tube occurs for 10-minutes. After the stimulation sequence is finished, the tube is removed, and the individual is instructed to immediately reproduce the sequence described above using the traditional EV acquisition techniques described in the literature, previously trained by the speech-language pathologist, which could be the aspiration, injection, or swallowing technique for 20-minutes. The stimulation of the pharyngoesophageal segment was performed once a week for three consecutive months, totaling 12-sessions.

## Results

Fifteen individuals were included: 14 were men and one was a woman, with a mean age of 54-years (ranging from 44- to 69-years). They had been disease-free for 4- to 10.4-years (mean of 7.6-years).

The VFS examination performed before the intervention demonstrated that all subjects presented good esophageal mobility during swallowing, but none presented opening of the pharyngoesophageal segment during simulated phonation. After the 12-sessions of esophageal stimulation, 9 (60%) of the subjects showed opening of the pharyngoesophageal segment during phonation.

The results of the Global Voice Assessment Protocol are presented in Table 1 and indicate that 9 (60%) of the subjects were able to produce EV of good or moderate quality.

**Table 1:** Results of the global voice evaluation before and after esophageal stimulation.

Vocal Quality	Pre-esophageal stimulation n (%)	Post-esophageal stimulation n (%)
Good	0	4 (27%)
Moderate	0	5 (33%)
Poor	15 (100%)	6 (40%)
Total	15 (100%)	15 (100%)

The auditory-perceptual evaluation of vocal quality, performed by the three speech-language pathologist judges pre- and post-esophageal stimulation, indicated that the majority of the judges perceived vocal quality as “poor” at the pre-stimulation moment, and there was a slight improvement in the proportion of poor responses at the post-evaluation moment, although it was not statistically significant. For analysis, the variables “Good”, “Moderate”, and “Poor” were converted into numerical ordinal variables: 1- Good, 2- Moderate, 3- Poor.

**Fig. 4:**
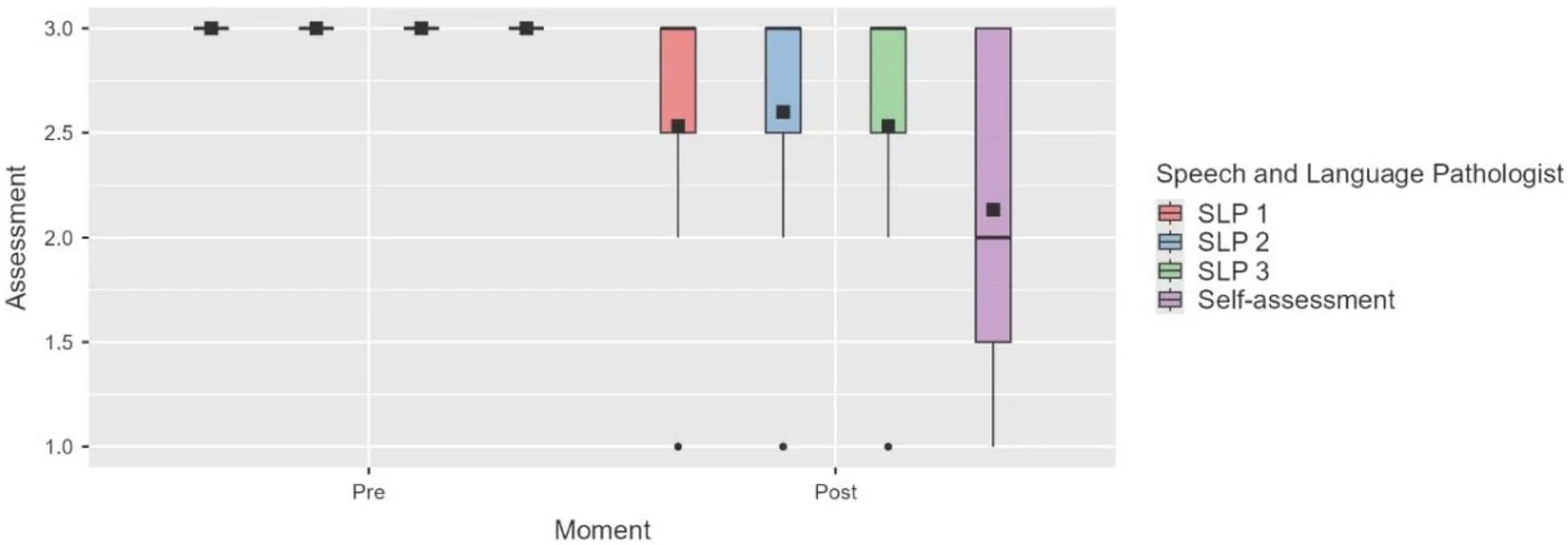
Descriptive analysis of the speech-language evaluations and self-assessment pre- and post-stimulation.

At the Pre moment, the mean of the three speech-language pathologists' evaluations was 3 (poor), with no variation in responses (standard deviation = 0). At the Post moment, the mean scores decreased to values ranging from 2.53 to 2.13. The confidence intervals range between 1.67 and 3.00. This indicates that there was greater variability in the responses, reflected in the standard deviation (0.84 and 0.73). The descriptive analysis of the speech-language evaluations and the participants' self-assessment can be observed in Fig. 4.

To compare the proportions of participants who showed improvement between the pre- and post-stimulation moments, the two-sided binomial test was performed. The observed proportions of improvement are presented in Table 2. None of the results were statistically significant at the 5% level.

**Table 2:** Result of the proportion of the speech-language evaluation pre- and post-esophageal stimulation and of the participants' self-assessment.

	SLP 1		SLP 2		SLP 3		Self-assessment	
	Pre	Post	Pre	Post	Pre	Post	Pre	Post
Good	0	3	0	2	0	3	0	4
Moderate	0	1	0	2	0	1	0	5
Poor	15	11	15	11	15	11	15	6
Total	15	15	15	15	15	15	15	15
Improved	4 (26.7%)		4 (26.7%)		4 (26.7%)		9 (60%)	
Not improved	11 (73.3%)		11 (73.3%)		11 (73.3%)		6 (40%)	
95% CI	7.8%–55.1%		7.8%–55.1%		7.8%–55.1%		32.3%–83.7%	
p-value	0.118		0.118		0.118		0.607	

Bilateral binomial test; 95% CI, Confidence Interval.

Table 3 demonstrates the results of the Wilcoxon test, which was applied to verify if there are significant differences pre- and post-stimulation considering the speech-language evaluation and the patient's self-assessment. A statistically significant difference was observed only for the participants' self-assessment (W = 45, p = 0.007), with a slight decrease in the means when comparing the two moments; higher values indicate poor vocal quality (3) and lower values indicate good vocal quality (1).

**Table 3:** Comparison of results obtained pre- and post-stimulation of the speech-language evaluation and self-assessment in the pre- and post-moments using the Wilcoxon test.

		Wilcoxon’s W	p
Pre SLP 1	Post SLP 1	10^a^	0.089
Pre SLP 2	Post SLP 2	10^a^	0.095
Pre SLP 3	Post SLP 3	10^a^	0.089
Pre-self assessment	Post- assessment	45^b^	0.007^c^

a 11-pairs of tied values.

b 6-pairs of tied values.

c Statistical significance.

## Discussion

This study investigated the use of a device as a therapeutic resource in speech therapy that provides direct stimulation of the pharyngoesophageal segment and may favor the production of EV. The device used is well described in the literature, primarily related to the use of the tube for esophageal evaluation before voice prosthesis placement.^10^, ^11^, ^12^, ^13^, ^14^ This study provides a novel perspective by revisiting the use of EV, which has significantly decreased over the last 40-years.^4^

The main findings indicate that, despite the statistical analysis not showing statistically significant differences in the proportion of participants who showed improvement in the speech-language evaluation in the pre- and post-stimulation moments, a proportion of participants demonstrated clinical improvement. Previous research has reported that approximately 30% of patients are able to acquire esophageal voice using conventional approaches.^13^ In the same context, direct stimulation of the pharyngoesophageal segment through tracheoesophageal phonation has been associated with a reduction in the time required for acquisition, from approximately 184-days to 59-days.^13^

Although the intervention method in the present study differs, the proposed approach may involve similar mechanisms, such as direct stimulation of the pharyngoesophageal segment and immediate auditory feedback. However, differences in study design and outcome measures limit direct comparisons between studies. These findings suggest that similar facilitating effects may be achieved using this non-surgical approach.

The hypothesis for better EV production after 12-weeks of training is related to the greater volume of air in the esophagus provided by the tube, which favors the opening of the pharyngoesophageal segment. The inability to retain air and the consequent lower intra-esophageal pressure makes it difficult or prevents the learning of the EV by the methods previously described.^17^ Another hypothesis for a higher acquisition rate in a shorter stimulation time is the auditory and neurosensory feedback that the subject receives, which occurs from the first stimulation session, where it is possible to produce the EV.

In this context, EV rehabilitation traditionally involves different air intake techniques, including deglutition, injection, and suction, which are progressively trained to enable pharyngoesophageal vibration.^18^ These approaches require repeated practice, gradual automatization, and are influenced by factors such as motivation, anatomical conditions, and therapy intensity.^18^

Alternative strategies, such as biofeedback and structured behavioral programs, have been proposed to facilitate EV acquisition, although without clear superiority over conventional approaches.^7^^,^^19^ Despite these developments, variability in training methods and the lack of standardized protocols remain important challenges in EV rehabilitation.^20^

The developer of the device mentions in the original study that it can be used as a predictor for EV,^9^ but no other studies reporting this use were found. A hypothesis for this may be related to the fact that, in developed countries, the use of Tracheoesophageal Voice Prosthesis (TEVP) is a reality, and the routine of health services is different from what occurs in Brazil.

From a clinical perspective, the findings suggest that the insufflation tube may be used as a facilitating tool in speech-language pathology practice, particularly in the early stages of EV training. Its ability to provide immediate sound production and feedback may support patient engagement and understanding of the target vocal behavior. Additionally, its low cost and non-surgical nature make it a potentially accessible option in settings with limited access to tracheoesophageal prostheses, such as the Brazilian public health system.

The reality in Brazil, a country of continental size, is characterized by limited availability of TEVP, mainly restricted to large centers, and even then, with limitations. This being the case, the use of a device already developed for the purpose of EV production becomes necessary as a potential strategy to improve access to rehabilitation, in addition to becoming more accessible to the population.

The study participants, on the other hand, significantly self-assessed their vocal quality as good or moderate after stimulation, which diverged from the speech-language evaluation and may be related to the fact that the production they achieved may have been judged as poor by the professionals, who possibly adopted stricter evaluation criteria.^21^ However, this production may have been capable of promoting beneficial effects on subjective aspects related to the participants’ communication, as well as increasing self-perception, with kinesthetic effects.^22^ The expectation of improvement through training, as well as engagement, may also have favored greater self-perception of progress among the participants.^23^ The undeniable role of self-assessment is highlighted as one of the pillars of multidimensional voice evaluation, which encompasses both objective and subjective changes related to sensations and quality-of-life effects.

It is important to highlight that all participants who developed EV and were awaiting TEVP placement declined the desire to communicate with TEVP, exclusively using EV. Furthermore, the findings related to VFS are notable, which, although descriptive, point to a greater opening of the pharyngoesophageal segment.

Success in EV acquisition involves diverse processes and is highly dependent on the subject's ability to learn and execute new tasks, which decreases with advancing age.^24^ These factors are potentially minimized with the use of the resource presented in this study, given that there is a direct and objective stimulus in the esophagus, which does not require comprehension of the execution.

This device proved to be a potentially promising resource in the rehabilitation of the total laryngectomized individual, also having other positive aspects, such as low cost, which does not burden health services, in addition to being a facilitating resource in speech-language pathology practice. The design of the present study was elaborated to prospectively reflect a real-life treatment scenario. The results obtained should be interpreted with caution due to the limitations imposed by the study design.

Despite efforts to recruit the largest possible number of participants, strict inclusion criteria were applied to ensure sample homogeneity. As a result, the included participants may not fully represent the broader population of laryngectomized individuals. Also, the number of included subjects was reduced, which hampered sample randomization processes and the creation of a control group, which are considered limitations of the study, in addition to the fact that the intervention group was its own control group.

Furthermore, intra- and inter-observer reliability were not formally assessed, as repeated samples were not included for this purpose, which may have impacted the consistency of the auditory-perceptual evaluations. In addition, the vocal quality assessment protocol used in this study was translated into Brazilian Portuguese but has not undergone formal validation, which may affect the robustness of the perceptual findings.

In addition, the relatively short intervention period (12-sessions) may limit the understanding of long-term outcomes and sustainability of esophageal voice acquisition. Furthermore, the present study did not include a detailed characterization of participants. A more comprehensive analysis of clinical, anatomical, and demographic factors (such as age, comorbidities, or functional limitations) may help define eligibility criteria and optimize rehabilitation strategies, as well as improve the understanding of factors associated with both successful and unsuccessful outcomes. This aspect should be further explored in future studies.

To overcome the limitations of the present study, such outcome measures should be used in a randomized controlled clinical trial comparing the esophageal stimulation technique and the conventional EV rehabilitation technique. Therefore, the results should be interpreted with caution, as they may not be directly generalizable to other populations without further validation in larger and more diverse cohorts. Future studies with larger samples, controlled designs, and validated assessment instruments are needed to further investigate the effectiveness of this approach. Despite these limitations, the findings suggest that the proposed intervention is a potentially feasible and clinically relevant alternative for facilitating esophageal voice acquisition.

## Conclusion

Esophageal stimulation with the device described in this study was associated with improvements in patients’ self-assessment and enabled a proportion of subjects to achieve functional esophageal voice. The device may represent a feasible and accessible therapeutic resource for voice rehabilitation after total laryngectomy. However, these findings should be interpreted with caution, and further controlled studies are needed to confirm its effectiveness and to better define its role in clinical practice.

## ORCID ID

Ana Carolina Constantini: 0000-0002-0387-5166

## Funding

This study was supported by the Coordenação de Aperfeiçoamento de Pessoal de Nível Superior (CAPES), Brazil – Finance Code 001.

## Data availability statement

The authors declare that all data are available in repository.

## Declaration of competing interest

The authors declare no have conflicts of interest.
