# Supplementary material for: Therapeutic device as a facilitating resource for esophageal voice acquisition
Source: Braz J Otorhinolaryngol. 2026 Sep 10;92(6):101893. doi: 10.1016/j.bjorl.2026.101893 (PMC13586567; doi:10.1016/j.bjorl.2026.101893)
Supplement: Supplementary file 1 [file mmc1.docx]

BJORL-D-25-00377_Supplementary Material

**Annex 1** Global voice assessment form.

|  | **Good** | **Moderate** | **Poor** |
| --- | --- | --- | --- |
| **1.** Syllables per breath |  |  |  |
| **2.** Maximum phonation time /a/ |  |  |  |
| **3a.** Loudness variation |  |  |  |
| **3b.** Maximum loudness |  |  |  |
| **4.** Ease of phonation |  |  |  |
| **5.** Articulation |  |  |  |
| **6.** Modulation |  |  |  |
| **7.** Speaking rate |  |  |  |
|  |  |  |  |
| **1.** Stoma noise |  |  |  |
| **2.** Inspiratory audibility |  |  |  |
| **3.** Redundant movements |  |  |  |
|  |  |  |  |
| **1.** Voice quality |  |  |  |
| **2.** Intelligibility |  |  |  |
|  |  |  |  |
| **Final result:**________________ |  |  |  |

HILGERS, F. J. M.; AALDERS, M.; KERREBROEK, J. M.; et al. The Provox System: A Practical Guide to Postlaryngectomy Vocal and Pulmonary Rehabilitation. Amsterdam: INCA Medical Publishing, 1995.
